# The Complex Management of Atrial Fibrillation and Cancer in the COVID-19 Era: Drug Interactions, Thromboembolic Risk, and Proarrhythmia

**DOI:** 10.1007/s11897-020-00485-9

**Published:** 2020-10-06

**Authors:** Milo Gatti, Emanuel Raschi, Elisabetta Poluzzi, Cristian Martignani, Stefania Salvagni, Andrea Ardizzoni, Igor Diemberger

**Affiliations:** 1 Department of Medical and Surgical Sciences, Alma Mater Studiorum - University of Bologna, Bologna, Italy; 2 Cardiology Unit, Department of Experimental, Diagnostic and Specialty Medicine, Alma Mater Studiorum - University of Bologna, Bologna, Italy; 3 Division of Oncology, S. Orsola-Malpighi Hospital, Bologna, Italy; 4 Medical Oncology Unit, Department of Experimental, Diagnostic and Specialty Medicine, Policlinico S. Orsola-Malpighi, Alma Mater Studiorum - University of Bologna, Bologna, Italy

**Keywords:** Anticancer agents, Direct oral anticoagulants, Atrial fibrillation, QT prolongation, COVID-19, Drug-drug interactions

## Abstract

**Purpose of Review:**

Cardiotoxicity by anticancer agents has emerged as a multifaceted issue and is expected to affect both mortality and morbidity. This review summarizes clinical challenges in the management of oncological patients requiring anticoagulants for atrial fibrillation (AF) also considering the current outbreak of the COVID-19 (coronavirus disease 2019) pandemic, since this infection can add challenges to the management of both conditions. Specifically, the aims are manyfold: (1) describe the evolving use of direct oral anticoagulants (DOACs) in AF patients with cancer; (2) critically appraise the risk of clinically important drug-drug interactions (DDIs) between DOACs and oral targeted anticancer agents; (3) address expected DDIs between DOACs and candidate anti-COVID drugs, with implications on management of the underlying thrombotic risk; and (4) characterize the proarrhythmic liability in cardio-oncology in the setting of COVID-19, focusing on QT prolongation.

**Recent Findings:**

AF in cardio-oncology poses diagnostic and management challenges, also due to the number of anticancer drugs recently associated with AF onset/worsening. Oral targeted drugs can potentially interact with DOACs, with increased bleeding risk mainly due to pharmacokinetic DDIs. Moreover, the vast majority of oral anticancer agents cause QT prolongation with direct and indirect mechanisms, potentially resulting in the occurrence of torsade de pointes, especially in susceptible patients with COVID-19 receiving additional drugs with QT liability.

**Summary:**

Oncologists and cardiologists must be aware of the increased bleeding risk and arrhythmic susceptibility of patients with AF and cancer due to DDIs. High-risk individuals with COVID-19 should be prioritized to target preventive strategies, including optimal antithrombotic management, medication review, and stringent monitoring.

## Background

In parallel with the advancements of chemotherapy, targeted therapy, immunotherapy, and radiotherapy, cardio-oncology has become a recognized medical specialty and clinicians are increasingly facing the multifaceted spectrum of cardiovascular toxicities by anticancer agents, with risk stratification, prevention, and early recognition being major emerging challenges [1••, 2].

Cardiovascular toxicity with anticancer drugs is a rapidly evolving topic since it appears that no anticancer drug is fully devoid of cardiovascular liability. Historically, the original focus of cardio-oncology was on cardiotoxicity of old-fashioned chemotherapeutics, namely, delayed irreversible left ventricular ejection fraction impairment by anthracyclines, more recently shifting to targeted therapy, including reversible myocardial dysfunction by trastuzumab [3, 4]. With the advent of third-generation targeted therapy, including immunotherapy, venous thromboembolism, arterial toxic effects, myocarditis with immune checkpoint inhibitors, and cardiac dysfunction in the setting of cytokine release syndrome with chimeric antigen receptor T cell therapy have gained greater recognition and are receiving attention due to severity and high fatality rates. This is particularly relevant in view of the improved survival gained by oncologic patients after the introduction of these agents [5, 6].

In the oncological setting, the use of antithrombotic drugs, especially direct oral anticoagulants (DOACs), is expected to substantially increase in the near future for different reasons, namely, improved patient survival; the high prevalence of drug- and cancer-associated thrombosis; the intertwined relationship between cancer and atrial fibrillation (AF), including the emerging case of drug-induced AF by anticancer drugs; and the ongoing coronavirus disease 2019 (COVID-19) pandemic that poses further challenges in this intricate scenario, including proarrhythmia due to multiple QT-prolonging mechanisms. Clinicians should balance the therapeutic benefit versus the theoretical risk of drug-drug interactions (DDIs) to achieve the optimal safe prescribing.

Therefore, this review is aimed at (1) summarizing the evolving use of DOACs in AF patients with cancer; (2) critically appraising the risk of clinically important DDIs between DOACs and oral targeted anticancer agents; (3) addressing expected DDIs between DOACs and candidate anti-COVID drugs, with implications on management of the underlying thrombotic risk; and (4) characterizing the proarrhythmic liability in cardio-oncology in the setting of COVID-19, focusing on QT prolongation.

## Evolving Use of DOACs in AF Patients With Cancer: an Update

There is a close relationship between cancer and AF. In the ORBIT-AF registry, history of cancer was present in about a quarter of the 9749 patients analyzed. In particular, in 57%, it was a solid cancer, while in 1.3%, it was leukemia and in 3.3% a lymphoma. Notably, these patients presented a higher mortality coupled with increased risk of major bleedings [7]. Several studies confirmed the association between AF and cancer. In particular, the onset of AF was associated with a 2.5-fold increased absolute risk of a new diagnosis of cancer within the following 3 months (over 5 times greater than non-AF patients) [8], while in patients with a new diagnosis of cancer, there was a concomitant new diagnosis of AF in 2.4%, with doubled thromboembolic risk and a 6 times greater risk of heart failure [9]. Interestingly, this reciprocal interference was confirmed by a specific subanalysis of the Women’s Health Study [10]. Focusing on clinical outcomes, the ORBIT-AF registry and the ARISTOTLE trial did not highlight a different thromboembolic risk in patients with cancer with respect to the others; however, the number of incident active cancers was low, while in many patients, there was a history of cancer [11, 12]. Interestingly, focusing into de novo diagnosis of cancer or relapses in the ENGAGE AF-TIMI 48 study, Fanola et al. [13] confirmed overall a similar thromboembolic risk with respect to the remaining population but with an excess of events in patients with solid cancer vs. hematologic/dermatologic neoplasia (HR, 3.92; 95% CI, 121–127). Noteworthily, in all these studies, enrolled patients were candidates to DOACs in view of a CHADSVASC ≥ 2. However, looking at low-grade CHADSVASC scores (i.e., 0 [1••]), patients with cancer had an increased risk of both thromboembolic and bleeding events, according to the Danish registry [14]. As such, it remains unclear which scoring system (CHA2DS2-VASc or CHADS2) is preferable in the cancer population [15].

The introduction of DOACs for thromboembolic prophylaxis in AF patients with active cancer was hampered by the known thrombophilic/coagulopathic state of active malignancy and exclusion of known cancer patients from DOAC pivotal trials. Available evidence is in favor of DOACs also in this setting, but it derives from subanalysis and observational studies. In the ARISTOTLE trial, apixaban offered greater protection than warfarin in the primary endpoint among 1236 patients with a history of active/prior cancer [11]. In the ENGAGE AF-TIMI 48 trial, edoxaban showed significant improvement in the composite efficacy endpoint versus warfarin among 1153 patients with newly diagnosed cancer or disease recurrence [13]. Beyond these analyses, there are several observational studies confirming superiority vs. warfarin, but a proper trial is still lacking. It is also important to acknowledge that while low–molecular weight heparin (LMWH) is often a preferred anticoagulant to treat deep vein thrombosis/pulmonary embolism (DVT/PE) in cancer patients, there are no trials assessing the long-term efficacy and safety of LMWH in patients with AF to prevent thromboembolism [16]. In this regard, it is interesting to mention the ongoing Edoxaban for the Treatment of Coagulopathy in Patients With Active Cancer and Acute Ischemic Stroke (ENCHASE) study (Clinicaltrials.gov number: NCT03570281), which will compare edoxaban with LMWH for secondary prophylaxis in cancer-related stroke patients. This is relevant since despite the common use of LMWH in these patients, it has to be remembered that their use in acute stroke should be avoided [17••].

Thrombocytopenia and renal failure are comorbidities commonly found in cancer patients, which decrease the safety margin of DOACs and in general for anticoagulation. Similar to the assessment of thromboembolic risk, there is no score for estimating bleeding risk in cancer patients, and the widely adopted (despite the recognized limitations) HAS-BLED score does not incorporate thrombocytopenia. Moreover, pre-approved DOAC trials excluded patients with platelet values < 90,000–100,000. Regarding renal failure, it is known that its prevalence is frequent among patients with active cancer (e.g., in general, a GFR < 60 ml/min is present in > 15% of these subjects) and up to 80% of these patients are treated with nephrotoxic drugs [18]. Notably, acute kidney injury is recognized as a major complication of several chemotherapy treatments (affecting overall mortality), but suboptimal dose tailoring is frequent also in view of the limitations of the formulas to estimate renal function in these particular settings [19, 20]. For all these reasons, use of DOACs in cancer patients has to be considered also for thromboembolic prophylaxis in case of AF but this treatment should entail more frequent check for platelet counts, hemoglobin, and renal function than in standard AF population.

## Drug-Drug Interactions Between Targeted Anticancer Agents and Direct Oral Anticoagulants: a Bidirectional Concern?

As anticipated, the close relationship between cancer and pre-existing or new-onset AF [21] likely results in concomitant use of anticancer agents and DOACs, thus posing different concerns regarding occurrence of clinically relevant DDIs.

Although DOACs have minimal effects on the expression or activity of transporters or key CYP450 enzymes, actual impact on pharmacokinetic (PK) of anticancer agents is not expected; they are substrates for P-glycoprotein (P-gp) and therefore are all susceptible to agents modulating P-gp. Furthermore, all DOACs undergo liver metabolism, although CYP-dependent pathways are involved only for rivaroxaban and apixaban [22]. Consequently, the theoretical risk of interactions (mainly metabolic) appears to differ among DOACs as follows: rivaroxaban > apixaban > dabigatran ≈ edoxaban [23]. A summary of potential DDIs between DOACs and anticancer agents is shown in Table 1, where only oral targeted drugs are presented considering the role of the aforementioned metabolic liability as the main PK mechanism of interaction.

**Table 1:**
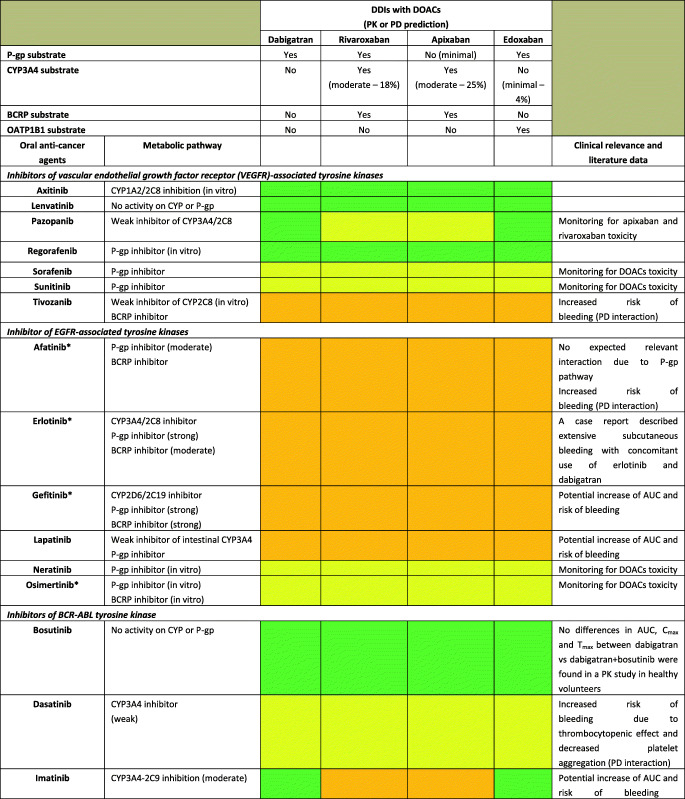

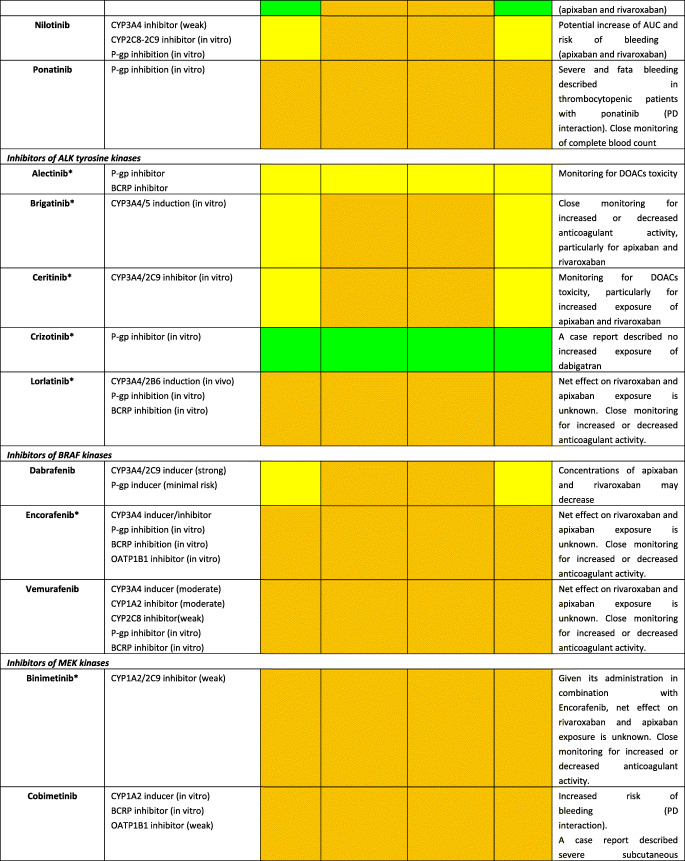

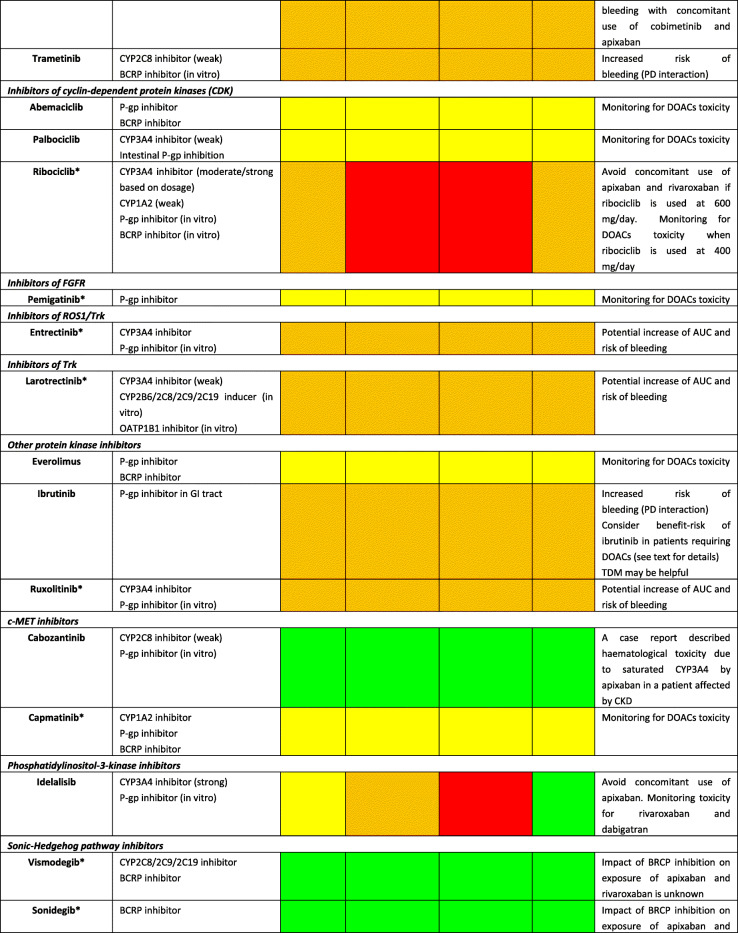

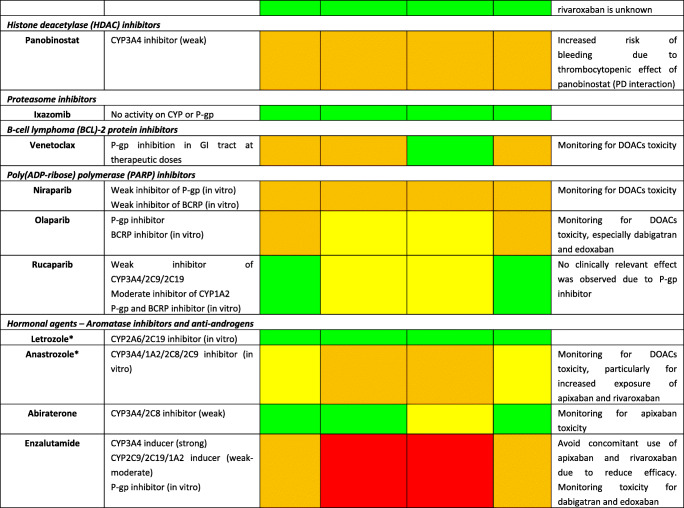
Predicted pharmacokinetic drug interactions between main oral anticancer agents and direct oral anticoagulants

*CYP* cytochrome P450, *P-gp* P-glycoprotein, *BCRP* breast cancer resistance protein, *AUC* area under the time-concentration curve, *PD* pharmacodynamic, *PK* pharmacokinetic, *TDM* therapeutic drug monitoring, *CKD* chronic kidney disease, *DOAC* direct oral anticoagulant, *DDI* drug-drug interaction, *GI* gastrointestinal, *OATP1B1* organic anion transporting polypeptide 1B1, *C*_*max*_ peak concentration, *T*_*max*_ time taken to reach peak concentration

Red box: avoid co-administration (contraindicated or not recommended). Orange box: potential interaction (caution should be exercised and consider dose adjustment or alternative drugs). Yellow box: potential weak interaction (monitoring for potential underexposure or toxicity). Green box: no interaction expected based on pharmacokinetic properties, although no clinical data exist

DDIs were checked through cancer-druginteractions.org, except for *, where DDIs were predicted on the basis of PK data retrieved from the summary of product characteristics or literature review

Among several potential DDIs occurring between DOACs and oral anticancer agents, only the concomitant administration of apixaban and idelalisib should be avoided, according to the specific online tool (https://www.cancer-druginteractions.org/), due to the strong inhibitory effect of idelalisib on CYP3A4 activity [24], although no real-world data exist. Furthermore, different anticancer agents show moderate/strong inhibition (namely, erlotinib, gefitinib, lapatinib, imatinib, nilotinib, ceritinib, ribociclib, ibrutinib, ruxolitinib, venetoclax, olaparib, capmatinib, entrectinib, and letrozole) or induction (namely, brigatinib, lorlatinib, dabrafenib, vemurafenib, and enzalutamide) activity on CYP3A4 or P-gp, potentially leading to anticoagulant over- or undertreatment (Table 1). Particularly, ribociclib exhibits strong inhibitory effect on CYP3A4 when used at high dose (600 mg/day); thus, concomitant use of apixaban or rivaroxaban should be avoided due to potential risk of severe anticoagulant overexposure [25]. Conversely, concomitant use of apixaban or rivaroxaban and enzalutamide (strong CYP3A4 inducer) should be avoided, considering the expected associated risk of anticoagulant underexposure [26]. Although PK/PD monitoring is not required for DOACs in clinical practice, and no trials support this approach, the aforementioned selected scenarios might be considered to assess the actual need for therapeutic drug monitoring. Clinicians should be reminded on the importance of critically reading the summary of product characteristics (SPCs) and publicly available online tools on the risk of DDIs (https://www.cancer-druginteractions.org/).

To the best of our knowledge, PK studies or case reports exploring clinical relevance of these DDIs between DOACs and anticancer agents are available only for dabigatran with erlotinib [27], bosutinib [28], ibrutinib [29–31], and crizotinib [32] and for apixaban with ibrutinib [33, 34], cobimetinib [35], and cabozantinib [36]. To address the discrepancy between theoretical pharmacological basis and actual clinical impact, there is urgent need to exploit big data such as pharmacovigilance and healthcare databases in identifying and prioritizing relevant DDIs. Moreover, pre-clinical studies present the limitation to investigate the interaction between single agents while it is common practice to have multiple drug schemes, including potential concomitant administration of multiple chemotherapies, antibiotics, antiarrhythmics, and even antiplatelet agents. These combinations create a specific milieu which is hard to face in view of the multiple cross-reactions as pointed out in simpler settings by the European Society of Cardiology consensus document on DOAC use [17••]. The concept rising from these considerations is the presence of a “coagulation reserve,” namely, an individual equilibrium between thrombotic and antithrombotic determinants at a certain time, affected by several concomitant factors (i.e., age, polypharmacy, underlying diseases, and organ failure), similar to the “repolarization reserve” present for the risk of development of torsade de pointes (TdP) [37], making clinically relevant a DDI also in cases where a relevant interaction is not expected according to pharmacological prediction.

Additionally, clinicians should be aware that also pharmacodynamic (PD) interactions may result in clinically relevant outcomes, considering that different anticancer agents increase per se the risk of severe bleeding (namely, tivozanib, afatinib, ponatinib, cobimetinib, trametinib, ibrutinib, panobinostat, encorafenib, and binimetinib association); in frail elderly cancer patients exposed to polypharmacotherapy, tight monitoring and risk minimization strategies should be performed, including medication review. Because DOACs do not require PK/PD monitoring and there are no biomarkers validated in clinical practice, patients should be alerted for early signs of minor bleedings, particularly when several agents (i.e., antibiotics, antiplatelet drugs, and chemotherapy) are concomitantly administered, given the potential synergic or exponential effect on occurrence of relevant DDIs.

## Direct Oral Anticoagulants in the Setting of COVID-19 and Cancer: a Double-Edge Sword

The real impact of polypharmacy and DDIs in frail patients affected by COVID-19 concomitantly treated with novel anticancer drugs and DOACs represents a current unmet clinical need. In addition to underlying conditions (cancer, AF), direct (i.e., hemostatic abnormalities caused by SARS-CoV-2 [38]) and indirect effects (i.e., intensive care unit [ICU] admission, deep sedation, mechanical ventilation, and potential prothrombotic action of agents used for the management of the infection) of COVID-19 call for an effective anticoagulation.

Several agents have been proposed for the management of COVID-19, including antivirals (e.g., remdesivir), antibiotics (azithromycin), and immunomodulators/immunosuppressants (e.g., hydroxychloroquine and tocilizumab) [39], although uncertainty about their efficacy in COVID-19 exists. Furthermore, different agents may exhibit PK and/or PD issues when concomitantly administered with DOACs, as reported in Table 2.

**Table 2:**
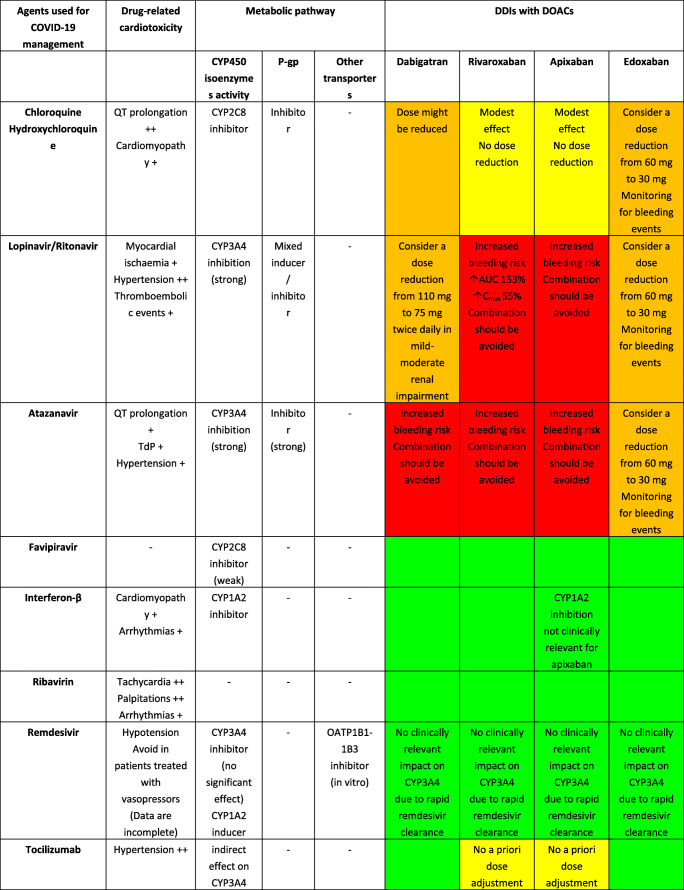

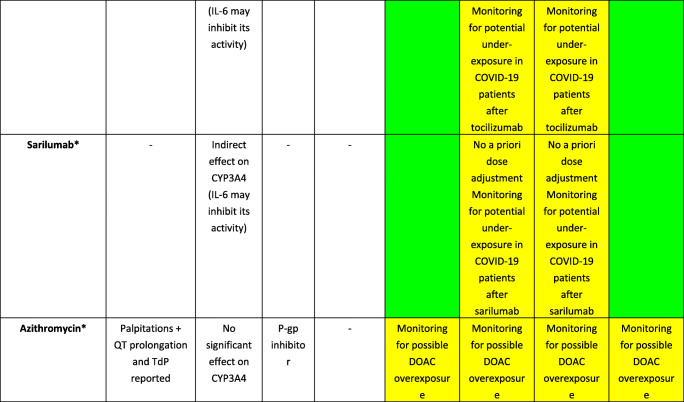
Key drug interactions and expected toxicities among direct oral anticoagulants and candidate drugs for COVID-19 management. Evidence concerning cardiotoxicity associated with agents used for COVID-19 management is also provided

*CYP* cytochrome P450, *P-gp* P-glycoprotein, *AUC* area under the time-concentration curve, *DOAC* direct oral anticoagulant, *DDI* drug-drug interaction, *OATP1B1* organic anion transporting polypeptide 1B1, *C*_*max*_ peak concentration, *TdP* torsade de pointes, *IL-6* interleukin-6

Red box: avoid co-administration (contraindicated or not recommended). Orange box: potential interaction (caution should be exercised and consider dose adjustment or alternative drugs). Yellow box: potential weak interaction (monitoring for potential underexposure or toxicity). Green box: no interaction expected based on pharmacokinetic properties, although no clinical data exist

DDIs were checked through https://www.covid19-druginteractions.org/, except for *, where DDIs were predicted on the basis of PK data retrieved from the summary of product characteristics or literature review

^++^Occurs frequently. ^+^Occurs occasionally according to summary of product characteristics

Notably, although several studies and case reports [40–47] investigated the impact of ritonavir or tocilizumab on DOAC exposure, none of these was performed in the context of COVID-19. Particularly, the degree of COVID-19 severity and associated renal and/or hepatic injury may increase the likelihood and clinical relevance of DDIs. In this regard, dysregulated immune systems coupled with cytokine storms, mainly involving overproduction of interleukin-6 (IL-6), are important causes of death in severe COVID-19 [48]; thus, tocilizumab and sarilumab, acting as antagonists of the IL-6 receptor, have been proposed for the management of critically ill patients [37, 47]. However, elevated IL-6 levels were found to suppress activities of CYP3A4, CYP2C19, CYP1A2, and P-gp [49, 50], and treatment with tocilizumab or sarilumab led to restoration of enzyme activity to non-disease levels [40, 51, 52]. Consequently, caution should be paid in COVID-19 patients treated with IL-6 receptor antagonists and concomitant DOACs (i.e., apixaban or rivaroxaban) and anticancer agents (i.e., ibrutinib, ixazomib, sunitinib, and olaparib) extensively metabolized by CYP3A4, given that unpredictable changes in relevant drug exposure may occur. Furthermore, ritonavir and atazanavir, by virtue of their strong inhibition on CYP3A4 and P-gp, pose several concerns when concomitantly used with DOACs or anticancer agents metabolized through these pathways. Combination should be avoided in order to prevent life-threatening bleeding caused by anticoagulant overexposure, as recently demonstrated in patients treated with DOACs and lopinavir/ritonavir or darunavir/ritonavir in the COVID-19 setting [53].

In consideration of the PK properties of DOACs, the clinical impact of interactions due to the treatment of COVID-19, the possible necessity of ICU admission and mechanical ventilation in severe cases with potential impairment of DOAC absorption, and the requirement for effective anticoagulation, replacing oral anticoagulant therapies with parenteral LMWH or unfractionated heparin was suggested to avoid the risk of over/undertreatment [53, 54, 55••]. A dedicated online tool is available for safe prescribing in patients with COVID-19 (https://www.covid19-druginteractions.org/).

## Proarrhythmic Liability in Cardio-oncology: the Emerging Case of Atrial Fibrillation by Anticancer Drugs

The relationship between anticancer therapy and arrhythmias is not well established, and the arrhythmogenic mechanisms remain uncertain as they can be the result of a direct electrophysiological effect or indirect perturbation of cardiac structure and function, including myocardial ischemia and heart failure, which in turn create an arrhythmogenic substrate [56].

Anticancer drug–induced AF is attracting emerging concern and poses diagnostic challenges due to competing factors, including the twofold increased risk of AF by cancer per se and the increased incidence of post-operative AF after pulmonary resection [57, 58]. Data on iatrogenic AF are scarce and essentially based on non-randomized clinical trials, underpowered, and not sufficiently pragmatic to enroll and characterize real-world patients with cardiovascular risk factors or those having pre-existing cardiac diseases (i.e., highest risk of AF) [59].

A recent pharmacovigilance analysis of WHO VigiBase provided the largest characterization of anticancer drug–induced AF and found 19 agents with significant overreporting. Apart from confirming some widely known associations such as ibrutinib, 9 potentially novel associations were identified especially in hematologic malignancies, including immunomodulating agents (lenalidomide, pomalidomide), kinase inhibitors (nilotinib, ponatinib, and midostaurin), antimetabolites (azacitidine, clofarabine), docetaxel (taxane), and obinutuzumab, with consistent results across sensitivity analyses accounting for potential confounders (e.g., co-reporting of antiarrhythmic drugs and anticoagulants as proxies of pre-existing AF). Considering that anticancer drugs with the strongest associations affect the phosphoinositide 3-kinase/protein kinase B (PI3K/AKT) and mitogen-activated protein kinase (MAPK) signaling pathways, the authors hypothesized these pathways as mostly implicated in AF associated with anticancer drugs, as compared with the “classical” molecular pathway of calcium-handling abnormalities [60]. Clinicians should be aware that risk of AF is also associated with bisphosphonates, mainly zoledronic acid, frequently used in patients with bone metastasis [61].

In this scenario, AF is generally manageable without anticancer drug discontinuation, to minimize the risk of treatment failure: patients who had ibrutinib interrupted for AF onset had decreased progression-free survival (median 19 months) as compared with those who continued ibrutinib or had dose reductions (median 27 months, *p* = 0.023) [62]. The use of ibrutinib poses several challenges in cardio-oncology as multiple cardiovascular toxicities were recently described in a pharmacovigilance study using VigiBase, which found overreporting of severe and occasionally fatal cardiac events, including conduction disorders, supraventricular arrhythmias, heart failure, and hemorrhagic events, especially at the central nervous system [63].

Although no randomized clinical trial addressed the rate versus rhythm control strategies in cancer patients experiencing AF associated with anticancer drugs, lessons learned from the management of AF associated with ibrutinib or other drugs can serve as initial guidance [64]: in hemodynamically stable cancer patients with AF related to anticancer drugs, rate control may be preferable to rhythm control because the ability to maintain sinus rhythm after cardioversion may be limited by the continuation of the imputed anticancer drug. Another possibility for rhythm control is AF ablation, especially for patients with impaired left ventricular function. In this subgroup of patients, the CASTLE-AF trial [65] showed improved survival with respect to non-ablation strategy. However, AF ablation in patients with cancer has never been explored due to several issues: efficacy of standard pulmonary vein isolation (in view of possible different mechanisms in cancer patients) and thromboembolic and hemorrhagic risk especially in view of the unpredictable effects of the neoplastic condition on atrial lesions and on effectiveness of standard anticoagulation regimens in both the acute and long-term phases. Theoretically, adoption of cryoablation could provide the best safety margin in view of reduced thrombogenic lesions, although additional confirmation is needed [66]. In a small cohort of 21 cancer survivors undergoing AF ablation [67], the risk of clinically relevant bleedings at 30 days was 3.6 times higher as compared with that of non-cancer patients. This trend remained after propensity score–matched population, and it was independent from the type of anticoagulation. However, enrolled patients had no active cancer treatment and they included a limited number of cancer types (i.e., gastrointestinal, breast, and genitourinary) limiting transferability to other settings. The future evolution of AF ablation through introduction of non-invasive radiosurgery may provide a better approach for sinus rhythm maintenance in cancer patients [68]. Clinicians should be reminded that the decision to use anticoagulants in this setting remains challenging as both CHA2DS2-VASc and HAS-BLED scores were not validated in cancer patients. Moreover, as anticipated, DDI risks should not be overlooked and bleeding risk may be also related to the underlying malignancy, with gastrointestinal cancer more at risk [69].

## Proarrhythmic Liability in Cardio-oncology: Focus on QT Prolongation

Drug-induced QT prolongation is a recognized surrogate marker of cardiotoxicity, potentially leading to the so-called TdP, which may ultimately result in ventricular fibrillation and sudden cardiac death. Drug-induced TdP is still a research and clinical priority in 2020, although the focus shifted from the pre-marketing risk prediction [70] towards the post-marketing real-world setting [71].

The persisting need for bridging the gap between pathophysiological knowledge and clinical practice is demonstrated by the ongoing efforts of Arizona CERT to implement crediblemeds.org, an interactive website devoted to risk assessment of drug-induced QT prolongation [37]. Apart from the widely known section on QT drug lists, the online tool was recently supplemented with several features for healthcare providers [72]: (1) *QTFactors.org*, listing clinical risk factors associated with QT/TdP risk, also in terms of strength of the evidence (e.g., bradycardia is a strong risk factor for TdP, with high strength of the evidence); (2) *OncoSupport*, a dedicated printable list of QT-prolonging drugs that are prescribed in patients with cancer, including anticancer agents, antiemetics, antidepressants, and anesthetics, with relevant categorization for TdP liability; and (3) *MedSafetyScan*, a clinical decision support tool specifically released for real-time proarrhythmic risk assessment in the COVID-19 setting (https://medsafetyscan.org/index.php), offering customized scores depending on the setting (ICU vs. outpatients).

Several targeted anticancer agents such as tyrosine kinase inhibitors (TKIs) are associated with QT liability [73, 74], both via direct effect on ventricular repolarization and through indirect properties, such as heart failure (risk factors per se of TdP). This risk is frequently recognized in the SPCs, in terms of QT prolongation, generic arrhythmia, tachycardia/bradycardia, or indirectly by citing decreased LVEF or myocardial ischemia. Because of the still uncertain causal association, data from spontaneous reporting systems such as the FDA Adverse Event Reporting System (FAERS) should be routinely monitored and update of SPCs is recommended when cases suggesting proarrhythmic risk are increasing (Table 3). This seems for instance the case of axitinib, regorafenib, erlotinib, idelalisib, and venetoclax, for which many cases of AF have been reported in FAERS, without any warning in the relevant SPCs.

**Table 3:** Cardiotoxicity of different oral anticancer agents according to the summary of product characteristics and adverse reactions retrieved from the US Food and Drug Administration Adverse Event Reporting System (FAERS)*

Oral anticancer agent	Cardiotoxicity					Vascular events	FAERS data					
	Decreased LVEF	Arrhythmias	QT prolonged	Myocardial ischemia	Bradycardia Tachycardia		Overall AEs	Arrhythmic events			Predisposing events	
								AF	QT↑	TdP	Diarrhea	Vomiting
Inhibitors of VEGFR-associated tyrosine kinases												
Axitinib	++	0	0	0	0	↑HYP: +++ B: +++ TE: ++	7623	50	10	6	960	235
Lenvatinib	++	0	++	++	0	↑HYP: +++ B: +++ TE: ++	8116	36	31	0	1419	705
Pazopanib	+	0	+	+	BR: +	↑HYP: +++ B: + TE: ++	21,302	76	28	3	2982	1174
Regorafenib	0	0	0	+	0	↑HYP: +++ B: +++	6783	46	3	0	864	356
Sorafenib	++	0	+	++	0	↑HYP: +++ B: +++	18,454	167	29	5	2721	910
Sunitinib	++	0	+	++	0	↑HYP: +++ B: + TE: ++	34,994	147	69	10	4066	2079
Tivozanib	0	0	+	++	T: ++	↑HYP: +++ B: ++ TE: ++	60	1	0	0	3	2
Inhibitor of EGFR-associated tyrosine kinases												
Afatinib	0	0	0	0	0	B: ++	4789	18	2	0	1741	349
Erlotinib	0	0	0	0	0	B: ++	42,033	176	18	5	5123	1434
Gefitinib	0	0	+	0	0	B: ++	7016	47	22	3	804	289
Lapatinib	++	0	+	0	0	0	13,495	37	31	1	3966	1076
Neratinib	0	0	0	0	0	0	1210	0	0	0	745	180
Osimertinib	++	0	+	0	0	0	6132	56	80	11	468	113
Inhibitors of BCR-ABL tyrosine kinase												
Bosutinib	0	0	++	0	0	↑HYP: +++	4047	44	8	0	1216	300
Dasatinib	++	++	+	+	0	↑HYP: ++ ↓HYP: + B: +++ TE: +	21,050	141	90	2	1343	630
Imatinib	+	0	0	0	T: +	↑HYP: + ↓HYP: + B: ++	47,794	256	128	5	2265	1906
Nilotinib	+	++	++	0	0	↑HYP: ++ B: +	20,777	313	671	7	664	669
Ponatinib	++	++	0	++	0	↑HYP: +++ TE: ++	6006	105	15	0	176	175
Inhibitors of ALK tyrosine kinases												
Alectinib	0	0	0	0	BR: ++	0	1880	6	7	0	41	20
Brigatinib	0	0	++	0	BR: ++ T: ++	↑HYP: +++	692	2	1	0	35	19
Ceritinib	0	0	+	0	BR: ++	0	2006	11	21	0	361	205
Crizotinib	++	0	++	0	BR: +++	0	8841	46	78	0	651	563
Lorlatinib	+	+	+	0	BR: +	0	710	2	8	0	17	7
Inhibitors of BRAF kinases												
Dabrafenib	++	0	+	0	BR: +	↑HYP: +++ ↓HYP: ++ B: +++	10,748	83	54	0	456	478
Encorafenib	++	0	++	0	T: ++	↑HYP: +++ B: +++ TE: ++	1823	2	7	0	157	131
Vemurafenib	0	0	+	0	0	0	9449	71	107	3	654	386
Inhibitors of MEK kinases												
Binimetinib	++	0	++	0	T: ++	B: +++	1913	3	6	0	160	130
Cobimetinib	+	0	0	0	0	↑HYP: +++ B: +++	2443	28	31	0	250	112
Trametinib	++	0	0	0	BR: ++	↑HYP: +++ ↓HYP: ++ B: +++ TE: +	11,437	84	45	0	557	491
Inhibitors of cyclin-dependent protein kinases (CDK)												
Abemaciclib	0	0	0	0	0	TE: ++	3062	16	0	0	912	201
Palbociclib	0	0	0	0	0	0	39,646	97	52	1	2363	1396
Ribociclib	0	0	++	0	0	0	5210	64	252	1	315	300
Inhibitors of FGFR												
Pemigatinib	0	0	0	0	0	0	3	0	0	0	0	1
Inhibitors of ROS1/Trk												
Entrectinib	++	0	++	0	0	↓HYP: ++	61	0	0	0	1	0
Inhibitors of Trk												
Larotrectinib	0	0	0	0	0	0	129	1	0	0	4	3
Other protein kinase inhibitors												
Everolimus	0	0	0	0	0	↑HYP: +++ B: ++	35,421	154	31	1	2755	1497
Ibrutinib	0	++	0	0	0	↑HYP: +++ B: +++	32,166	1809	12	6	2183	572
Ruxolitinib	0	++	0	0	0	↑HYP: +++ B: +++	33,401	208	10	0	1454	665
c-MET inhibitors												
Cabozantinib	0	0	+	+	0	↑HYP: +++ B: + TE: ++	13,603	54	8	0	2686	677
Capmatinib	0	0	0	0	0	0	19	1	0	0	0	0
Phosphatidylinositol-3-kinase inhibitors												
Idelalisib	0	0	0	0	0	0	5909	90	0	0	706	160
Sonic hedgehog pathway inhibitors												
Vismodegib	0	0	0	0	0	0	4904	11	1	0	274	168
Sonidegib	0	0	0	0	0	0	261	3	0	0	12	11
Histone deacetylase (HDAC) inhibitors												
Panobinostat	0	++	++	+	0	↑HYP: ++ ↓HYP: +++ B: ++	1610	31	21	0	341	104
Proteasome inhibitors												
Ixazomib	0	0	0	0	0	TE: +	10,482	61	3	0	1095	430
B cell lymphoma (BCL)–2 protein inhibitors												
Venetoclax	0	0	0	0	0	0	12,767	218	5	0	523	213
Poly(ADP-ribose) polymerase (PARP) inhibitors												
Niraparib	0	0	0	0	T: ++	↑HYP: +++	6475	49	3	0	422	777
Olaparib	0	0	0	0	0	0	4327	13	3	0	156	243
Rucaparib	0	0	0	0	0	0	4887	11	2	0	511	524
Hormonal agents: aromatase inhibitors and antiandrogens												
Letrozole	++	++	0	+	T: +	↑HYP: +++ TE: +	17,376	179	156	1	874	618
Anastrozole	0	0	0	++	0	TE: ++	14,877	88	12	1	433	255
Abiraterone	++	++	+	+	T: ++	↑HYP: +++	22,018	190	30	7	468	367
Enzalutamide	0	0	+	++	0	↑HYP: +++	42,170	197	47	1	2259	1012

*TE* thromboembolic events, *B* bleeding, *↑HYP* hypertension, *↓HYP* hypotension, *BR* bradycardia, *T* tachycardia, *QT↑* QT prolonged, *TdP* torsade de pointes, *AF* atrial fibrillation

*Data retrieved querying the public dashboard of the FDA adverse reporting system (FAERS; https://www.fda.gov/drugs/questions-and-answers-fdas-adverse-event-reportingsystem-faers/fda-adverse-event-reporting-system-faers-publicdashboard, searches performed on 14/05/2020; data as of March 31, 2020). It is important to quickly address limitations of this analysis, including data quality (potential existence of pre-marketing reports, duplicates, and missing information), the likelihood of underreporting, the potential influence of external factors (time on the market and media attention), the lack of exposure data (drug prescription/consumption), and the inability to establish firm causality, incidence, risk assessment, and risk ranking which cannot be provided. These data only provide a general picture of the current arrhythmic reporting pattern with novel oral anticancer agents

^+++^Occurs very frequently. ^++^Occurs frequently. ^+^Occurs occasionally. 0 not reported (according to the summary of product characteristics)

**Fig. 1:**
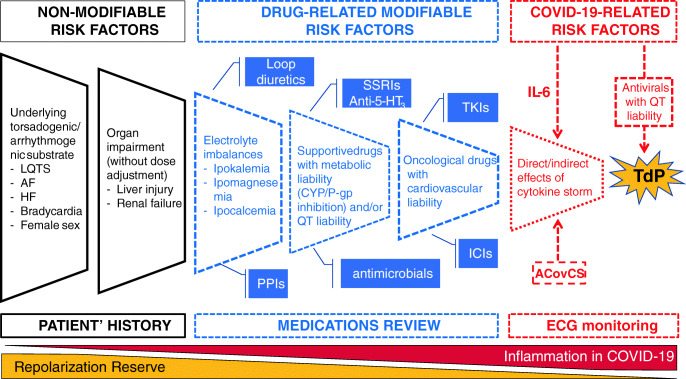
Revisiting the concept of the reduced repolarization reserve in cardio-oncology during the COVID-19 era. ACovCS, acute COVID cardiovascular syndrome; ICIs, immune checkpoint inhibitors; PPIs, proton pump inhibitors; SSRIs, selective serotonin re-uptake inhibitors; TKIs, tyrosine kinase inhibitors; LQTS, long QT syndrome; AF, atrial fibrillation; HF, heart failure; ECG, Electrocardiography

These effects may synergize with concomitant medications taken to counteract side effects of chemotherapy (e.g., antinausea) or treat comorbidities (e.g., antidepressants, antimicrobials, loop diuretics, and proton pump inhibitors), with potential PD/PK interactions (via cytochrome and/or P-gp inhibition), which in turn reduce the cardiac repolarization reserve with increased patients’ susceptibility to TdP (Fig. 1). In fact, the prevalence of potential DDIs with anticancer drugs may reach 78% in the real world [75], especially among targeted therapy, where QT prolongation is a common clinical consequence [76].

The blockade of the so-called hERG channel, which identifies the pore-forming alpha subunit of the rapid component of the delayed rectifier K+ current (IKr), is the most extensively studied and a key mechanism underlying drug-induced QT prolongation, although, at least theoretically, actions on other potassium currents may also account for a prolongation of the action potential duration [77]. Recently, the IKr-based paradigm was challenged by in vivo studies, which pointed out PI3K (alpha isoform in cardiomyocytes) as the major target mediating action potential prolongation by TKIs [78]. Lu et al. [79] described multiple mechanisms underlying action potential prolongation by PI3Ka inhibition: TKIs reduce IKr and IKs amplitude (which would prolong action potential), while reducing peak calcium and sodium current (expected to shorten the QT interval), thus explaining the arrhythmogenic potential of TKIs without acutely blocking IKr [80•]. This direct interaction with the hERG pharmacophore should rapidly result in QT prolongation, although also delayed onset of proarrhythmia was described with some anticancer drugs such as histone deacetylase (HDAC) inhibitors, which were reported to interfere with hERG channel trafficking [81]. Intriguingly, modifying the degree of selectivity towards HDAC isoforms might be a strategy to develop safer agents in terms of TdP liability: ricolinostat and citarinostat, HDAC6-selective inhibitors, theoretically possess better cardiac tolerability as compared with pan-HDAC inhibitors [82].

## Risk Stratification for Torsadogenicity: Myth or Reality?

According to crediblemeds.org, vandetanib, arsenic trioxide, aclarubicin, cesium chloride, and oxaliplatin are considered to carry torsadogenic risk in humans even when used as recommended, whereas several TKIs (e.g., osimertinib) are classified as “possible risk of TdP”; they can cause QT prolongation but there is insufficient evidence that these drugs, when used as directed in official labeling, are associated with a risk of TdP. Supportive medications with TdP proclivity are represented by ondansetron and domperidone and selective serotonin re-uptake inhibitors such as citalopram.

The critical challenge in all these scenarios is to identify safer therapeutic alternatives, for which clinical experience remains a crucial determinant. In fact, there is still ongoing debate on whether or not QT prolongation and TdP risk should be considered a class effect or peculiarities exist when prescribing within the same pharmacological class.

The case of TKIs that target the epidermal growth factor receptor (EGFR) is paradigmatic: apart from slight PK differences (with CYP3A4 being the main enzyme involved in the metabolism for a number of TKIs-EGFR), diarrhea should be considered a class effect of these drugs and may predispose to dehydration and hypokalemia, thus increasing the likelihood of TdP. Of note, hypomagnesemia appears to be a specific safety issue of osimertinib (occurring in up to 30% of patients), which may partially explain the higher frequency of post-marketing reports as compared with that of other anti-EGFR agents [83].

Diarrhea, nausea, and vomiting, which increase TdP susceptibility, are relatively common safety issues observed with different targeted agents, including with anaplastic lymphoma kinase (ALK) TKIs [84] and P13K inhibitors. Hepatocyte growth factor receptor (c-MET) inhibitors have a complex tolerability profile, with multiple mechanisms likely to result in proarrhythmia, including direct QT-prolonging effect (albeit rare), cardiac failure (rare instances especially described in the post-marketing setting), and dose-dependent symptomatic bradycardia (i.e., syncope, dizziness, and hypotension), especially with crizotinib observed in 2.4% of patients across clinical trials, and potentially developing after several weeks of drug initiation [85]. HDAC inhibitors are also expected to cause clinically significant QT prolongation although drug-specific peculiarities were observed in drug development and dedicated thorough QT studies were not systematically performed [86]. Among PI3K inhibitors, QT prolongation appears to be specific with copanlisib (intravenously administered), and a post-marketing study was committed by regulators to determine the proarrhythmic effect in subjects with advanced solid tumors and non-Hodgkin’s lymphoma [24]. Notably, unexpected reports of AF with idelalisib have been recently recorded in the EudraVigilance database, although their significance is unclear. Interestingly, ibrutinib (another small-molecule agent used to treat B cell lymphomas and leukemias) and idelalisib share a number of common safety issues [87]. Also, drugs targeting the hedgehog pathway are differently associated with QT prolongation, with arsenic trioxide carrying the highest risk, whereas vismodegib and sonidegib (inhibitors of Smoothened) do not appear to directly affect the QT interval, although diarrhea, nausea, and vomiting should not be overlooked as risk factors [88]. Cyclin-dependent kinase (CDK) 4/6 inhibitors and poly(ADP-ribose) polymerase (PARP) inhibitors are relatively new classes of anticancer drugs for which the evidence on proarrhythmic risk is still provisional, although QT prolongation was preferentially linked to ribociclib, and rucaparib was recently added in the list of drugs with “possible risk of TdP.”

In summary, QT prolongation should be taken as a class effect by the majority of anticancer drugs through direct or indirect mechanisms: direct hERG blockade/interference, P13K inhibition, PK interactions mediated by metabolic liability (CYP substrate/inhibition), and indirect PD effects (diarrhea, vomiting resulting in clinically important electrolyte imbalances such as hypokalemia and hypomagnesemia). However, the actual risk of TdP occurrence should be assessed in the individual patient and depends on the presence of multiple drug- and host-related risk factors, some of which are modifiable and minimized. Notably, post-marketing data presented in Table 3 suggested that TdP is only rarely reported with targeted anticancer drugs, as compared with QT prolongation, although the recent marketing approval of several agents and the likelihood of underreporting call for continuous pharmacovigilance monitoring, also in the light of high reporting of diarrhea and vomiting.

Therefore, as a general rule, electrocardiogram (ECG) represents an inexpensive, non-invasive monitoring tool to early recognize TdP and potentially prevent its occurrence through QT measurement, although a remarkable underuse was recently documented in oncology patients receiving QT-prolonging drugs [89]. Notably, we previously reported the results of a prolonged prospective ECG follow-up in a cohort of outpatients initiating chemotherapy in a university center specialized in female cancer. Enrolled patients were followed for 992 chemotherapy cycles (median 7; interquartile range 6–13). No cumulative effect on QTc prolongation nor a relevant effect of prophylactic/supportive drugs emerged. More interestingly, we defined a novel parameter, the baseline-first chemotherapy averaged QTc, based on measurement on baseline QTc and QTc after the 1st chemotherapy administration able to identify 100% of patients with Max-QTc > 500 ms and 96% with Max-QTc 481–500 ms during all the follow-up, independently to the type and number of chemotherapy regimens received [90]. This kind of approach should be further explored to identify the most cost-effective monitoring regimen for ECG monitoring in cancer patients.

## Torsadogenic Liability in Cardio-oncology: Focus on Patients With COVID-19 Infection

While our epidemiological knowledge about COVID-19 is rapidly evolving, emerging data are largely concordant in pointing out a systemic disease involving the liver, kidney, respiratory, inflammatory, immune, and cardiovascular system. The spectrum of the so-called acute COVID-19 cardiovascular syndrome encompasses a variety of manifestations, including coronary syndrome, myocardial injury without coronary artery disease, arrhythmias, thromboembolic complications, pericardial effusion, and de novo systolic dysfunction, with myocarditis/myopericarditis, cytokine storm, and stress-induced cardiomyopathy likely to be leading etiological factors of the proarrhythmic liability [91, 92]. Currently, a trial investigating the efficacy of early acute coronary syndrome therapy (including rivaroxaban) in preventing cardiac complication of COVID-19 disease is ongoing (Clinicaltrials.gov: NCT04333407).

Although it is currently believed that myocardial damage might represent a main driver of enhanced arrhythmic risk in COVID-19 patients, underlying mechanisms are still under investigation. Two main actors are likely to contribute: concomitant pharmacological treatments and high-grade systemic inflammatory state [93]. In cardio-oncology, these COVID-19 proarrhythmic factors may synergize with pre-existing arrhythmogenic substrates to reduce the repolarization reserve in the myocardium, with ultimate TdP onset (Fig. 1).

Different agents are used “off-label” to counteract virus invasion/replication and may promote QT prolongation. This is especially the case of chloroquine/hydroxychloroquine and lopinavir/ritonavir, which can impact ventricular repolarization via direct (hERG blockade) and indirect mechanisms (inhibition of CYP2D6 and CYP3A4, respectively). Of note, chloroquine/hydroxychloroquine has unique pharmacokinetics (no large impact of renal dysfunction, critical illness, or obesity is expected, although in critically ill patients, drug absorption might be hampered) and possesses multifunctional pharmacodynamic properties, including direct myocardial toxicity and bradycardia by modulating the hyperpolarization-activated current (If) [94]. Of note, the prophylactic use of chloroquine/hydroxychloroquine in predominantly healthy, asymptomatic healthcare workers, coupled with feasibility issues in performing ECG in the out-of-hospital setting, and the frequent combination with azithromycin (a potential QT-prolonging agent with in vitro activity against COVID-19) may further increase the likelihood of TdP occurrence, especially in subjects with unknown congenital long QT syndrome [95–97].

Fighting systemic inflammation in COVID-19 patients represents a promising therapeutic strategy in COVID-19, especially in severe cases. The so-called *inflammatory cardiac channelopathies* were described, as IL-6 was demonstrated to directly inhibit the hERG channel [98]. Moreover, inflammatory cytokines can induce cardiac sympathetic system hyperactivation, via the central hypothalamus-mediated (inflammatory reflex) and peripheral (left stellate ganglia activation) pathways [99], potentially resulting in arrhythmias especially in patients with inherited long QT syndrome. As anticipated, IL-6 inhibits CYP3A4, thereby modifying PK of several medications, including QT-prolonging drugs. Therefore, it seems rational to target inflammatory response to reduce cardiovascular complications and relevant arrhythmic events: tocilizumab causes rapid and significant QT shortening, with relevant decreases in cytokine and C-reactive protein levels [100]. In this perspective, it is interesting to recall the results of the Canakinumab Anti-inflammatory Thrombosis Outcome Study (CANTOS) trial [101]. In this study, the human monoclonal anti-human interleukin-1 beta antibody, canakinumab, reduced the rate of recurrent cardiovascular events in patients with previous myocardial infarction. Intriguingly, additional analyses revealed a dose-dependent trend towards reduction of hospitalization for heart failure (independent of prior history) and a lower risk of incident lung cancer. Notably, recent studies showed that IL-1β might have electrophysiological effects changing Ca^2+^ handling and cell-cell connection, being associated with the prolonged QTc interval and presence of AF, with preliminary results on protective effects on post-infarction patients [102, 103].

In this intricate scenario, there is no consensus on optimal management to mitigate the underlying torsadogenic substrate. Different guidelines and authors have proposed recommendations for QT monitoring [104–107]: although there is general agreement on the need for baseline QT assessment and general discontinuation rules (e.g., QT interval, corrected for cardiac frequency, > 500 ms, and increases > 60 ms), the timing for electrocardiographic re-assessment and re-check may vary depending on the drug (e.g., after 3–4 days of therapy initiation with hydroxychloroquine/azithromycin), as well as underlying expected susceptibility (e.g., in patients with borderline QT and structural heart disease, telemetry should be considered, also with wearable devices for out-of-hospital monitoring). Notwithstanding practicalities in carrying out home monitoring, timely correction of electrolyte imbalances before prescribing QT-prolonging agents (and monitoring relevant blood levels) is recommended, although it is unclear whether an empirical approach through preventive administration of potassium and magnesium is actually effective in mitigating the risk of TdP occurrence.

In summary, the burden of arrhythmogenicity in COVID-19 can be mitigated by optimizing concomitant pharmacotherapies: in the acute viral phase, medication review and cardiovascular monitoring remain cornerstones for safe prescribing especially in patients exposed to multiple QT-prolonging drugs, including anticancer drugs, whereas in the later systemic phase, IL-6 targeted therapies (tocilizumab and sarilumab, under investigation in clinical trials) can promote the recovery from multiorgan dysfunction although the actual impact on the (high) proarrhythmic risk remains unclear.

## Conclusion

The rapidly evolving spectrum of cardiovascular manifestations with novel targeted therapy poses new diagnostic and therapeutic challenges for oncologists and cardiologists, who must be aware of clinical pharmacology to identify clinically relevant DDIs. Considering the increasing use of DOACs in oncology, and the large number of anticancer drugs inhibiting the activity of CYP34 and/or P-gp predicted DDIs can result in overexposure to DOACs (for which no PK/PD monitoring is still required), thus increasing the risk of bleeding. The paucity of real-world data supports the need for active pharmacovigilance and pharmacoepidemiological research, especially for apixaban and rivaroxaban (partly metabolized by CYP3A4), as well as edoxaban, the latest approved DOAC.

Moreover, oncological patients are vulnerable to proarrhythmias, mainly due to multiple QT-prolonging agents, especially in patients with COVID-19, who frequently receive drugs with torsadogenic liability. These high-risk individuals should be prioritized to target preventive strategies, including optimal antithrombotic management, medication review, correction of electrolyte imbalances, and stringent ECG monitoring. Critical assessment of public online tools, including SPCs, is also crucial for safe prescribing.
